# The Institutional Treatment of Consumption

**Published:** 1916-06-03

**Authors:** 


### THE INSTITUTIONAL TREATMENT OF CONSUMPTION.

### III.—Gradation of Work and Exercise.*

One of the chief objects of sanatorium treatment is to accustom patients to an abundance of fresh air at all hours of the day or night. This will improve the appetite, quiet the nervous system, and tend to raise the resistance. Purer air also means less liability to secondary infection from air-carried bacteria. Indoors fresh air must be secured by open windows and cross-ventilation. What is popularly known as a draught cannot always be avoided, but by having windows widely open, good big currents of air circulate around the ward, and the small chilling streams which result when window-sashes are only lowered a few inches are avoided. Out of doors the process is automatic; the chief points to observe are to avoid dusty places, not to remain at rest if exposed to very cold winds, and not to get overheated by sitting directly in the sun if this is powerful.

Rest periods are governed by as strict a discipline as periods of exercise or graduated labour. As was seen in the routine time-table given, rest during one period of the day—that before dinner and after the morning's exercise or work—aims at being a very thorough and complete rest. No talked is allowed. Among certain classes of patients this ideal is not easy to maintain, and the trials of medical superintendents and sisters have by no means been lightened by the influx into the sanatorium world of large numbers of persons entering under the National Insurance Act or under County Council schemes.

### Rest-Chairs.

Out of doors when the weather is fine it is preferable to scatter the rest-chairs fairly widely to discourage sociability. In shelters or *Liegehallen*, where a roof and back wall protect from rain and wind, gossip and detrimental excitement are easily fostered, but such places have to be used sometimes. A certain number of small shelters for one or two patients are not uncommonly provided in sanatoria, but these are not numerous, and are used by special patients for resting in by day. In order to train a patient who is to be lent a shelter for use in his own garden when he gets home, a shelter may be used for sleeping in. The folding garden-chair of wood and canvas is probably the commonest rest-couch provided for ambulant patients. These chairs need to be broader and more strongly built than the cheap variety used in suburban gardens; when in use, they should be adjusted to the position approaching nearest the horizontal. A much better chair is one specially constructed with a foot-piece and leg-rest, not raised like the end section of a deck-chair, but sloping down nearly to the ground, from which it is raised by two small pram-wheels. These chairs are rigid, and cannot be folded up, so require considerable space for storage, but are easily moved about on the wheels. The ordinary cane deck-chair is also suitable.

After the preliminary probation days in bed the patient is usually allowed up on the first day for two hours in the ward; on the next day for four hours, and allowed to walk in the ward or on the verandah. When getting up for six hours the patient will be able to have dinner, as well as tea, with the others, and may be allowed one walk of a quarter to half an hour's duration. All being satisfactory as regards temperature, pulse, or symptoms, the time up is extended by steps until the patient is up all day; by this time most of the day should be spent out of doors and three half-hour walks performed. Walking is commenced on the flat at about two miles an hour. Going uphill may be attempted when the patient can walk two or three miles on the level. The gradients available must depend on the situation of the sanatorium, but graduated walking exercise can usually be easily arranged. It is well that the return journey should be downhill.

### Auto-Inoculation.

By thus gradually increasing the walking exercise till the patient can walk five miles without untoward symptoms he is prepared for undertaking graduated labour. The theory underlying graduated exercise is that known as auto-inoculation. Exercise will always be followed by a rise of temperature in consumptives just as in a healthy person; but in the former the rise will be greater and will last longer. It is a manifestation of the absorption by the patient of a dose of toxin, and the aim of the treatment is to ensure that only quite manageable amounts of toxin are set free as the result of exercise. Small doses are speedily dealt with by the system, and it is supposed that the resistance of the patient is thereby increased and the body becomes immunised by degrees. Therefore the effect of exercise on a patient must be carefully watched and estimated; a rise of temperature is to be expected, but need not be an indication for return to rest in bed. If after half-an-hour's rest the temperature returns to normal and there are no symptoms, such as headache or malaise, it may be assumed that the patient is not doing too much. A temperature over 100° persisting for an hour or two is an indication for rest—perhaps for a day only, or maybe for several days, according to the circumstances—and the patient must start afresh at the bottom of the scale, though he need not always go right back.

### Graduated Labour.

Graduated labour is now extensively employed in practically all sanatoria, though the facilities for developing good schemes must be governed by local conditions; in some institutions which were not specially built for use as sanatoria, the facilities for open-air work are neither suitable nor sufficient. At Frimley, graduated labour has been made a

* The previous articles appeared on May 6 and 20, 1916.

prominent feature for some ten years, and a somewhat rigid sorting out of patients has been the rule, in order to obtain a large proportion of those who were likely to benefit fully by the scheme. It will suffice to indicate by one example the nature of these schemes of graduated out-door labour in sanatorium grounds.

Grade I.—Picking up wood, potting plants, light weeding, carrying a basket of mould, watering plants, etc. The basket work is subdivided into three sections: in the first the load is about 12 lb., in the second 18 lb., and in the third 24 lb.

Grade II.—Using a small shovel for digging; cutting grass with a small machine; hoeing; chopping wood; sweeping; raking; painting.

Grade III.—Using a larger spade for digging or shovelling earth into a cart; sawing trees for firewood; mowing grass with a large machine; rolling lawns; using full-sized pitchfork.

Grade IV.—Working with pick and shovel; breaking ground; trenching; felling trees; planing wood; mixing concrete, etc.

The hours of work are four hours per day, or in some cases five hours. If possible, a man is put to work at his own trade while at the sanatorium; if he can stand this work for six hours a day he is presumed to be fit for discharge. This is as far as sanatorium treatment can be expected to go; but, of course, the conditions of the man's home and workplace are usually so inferior to those at the sanatorium that the handicap may soon mean a serious difference to his output and to his well-being.

For women, similar work is arranged, but smaller implements are used, and the heavier work is omitted. They are often put to work on a kitchen garden, and have charge of poultry. It is usual to provide a certain amount of indoor work for women, but only short spells of this are demanded—say, up to two hours. This work may also be graded on some such lines as follows:—

Grade I.—Dusting; cleaning brasses; folding up bed-quilts; washing and cleaning out lockers.

Grade II.—Sweeping wards; cleaning windows (no stretching beyond head-level allowed); washing cutlery and china; laying tables for meals; polishing.

Grade III.—Making beds; scrubbing floors; blackleading stoves; moving furniture; turning out cupboards.

Physical drill for half an hour a day is a form of exercise which should be encouraged, perhaps even more for the women than the men. Apart from the good effect it has on muscular tone, it is of great value in teaching them to expand properly the chest, and to ventilate all parts of the lungs in respiration.

Having provided sanatorium patients with food, rest, exercise, and work, it behoves the superintendent to pay some attention to the means of recreation. Boredom distinctly militates against progress, even under the best of conditions. In arranging for recreation it is advisable to confine it to certain fixed hours of the day, as its value is enhanced thereby. Quiet games of cards, chess, draughts, etc., may be permitted, and women may sew and play music. Singing is not allowable, as it unduly strains the lungs. Outdoor games for those sufficiently convalescent must be carefully supervised, and should at no stage be of a strenuous order, even "pat-ball" lawn-tennis being barred; this means that such games should be of the type exemplified by clock-golf, croquet, bowls.

